# Licorice (*Glycyrrhiza glabra*) Extracts-Suitable Pharmacological Interventions for COVID-19? A Review

**DOI:** 10.3390/plants10122600

**Published:** 2021-11-26

**Authors:** Joji Abraham, Singarayer Florentine

**Affiliations:** 1 School of Engineering, Information Technology, and Physical Sciences, Mt Helen Campus, Federation University Australia, Ballarat, VIC 3353, Australia; 2 Centre for Environmental Management, School of Science, Psychology, and Sport, Mt Helen Campus, Federation University Australia, Ballarat, VIC 3353, Australia; s.florentine@federation.edu.au

**Keywords:** ARDS, antiviral drug, anti-inflammatory drug, coronavirus, glycyrrhizic acid, glycyrrhetinic acid, medicinal plants, SARS-CoV-2 pandemic

## Abstract

Even though vaccination has started against COVID-19, people should continue maintaining personal and social caution as it takes months or years to get everyone vaccinated, and we are not sure how long the vaccine remains efficacious. In order to contribute to the mitigation of COVID-19 symptoms, the pharmaceutical industry aims to develop antiviral drugs to inhibit the SARS-CoV-2 replication and produce anti-inflammatory medications that will inhibit the acute respiratory distress syndrome (ARDS), which is the primary cause of mortality among the COVID-19 patients. In reference to these tasks, this article considers the properties of a medicinal plant named licorice (*Glycyrrhiza glabra*), whose phytochemicals have shown both antiviral and anti-inflammatory tendencies through previous studies. All the literature was selected through extensive search in various databases such as google scholar, Scopus, the Web of Science, and PubMed. In addition to the antiviral and anti-inflammatory properties, one of the licorice components has an autophagy-enhancing mechanism that studies have suggested to be necessary for COVID-19 treatment. Based on reviewing relevant professional and historical literature regarding the medicinal properties of licorice, it is suggested that it may be worthwhile to conduct in vitro and in vivo studies, including clinical trials with glycyrrhizic and glycyrrhetinic acids together with other flavonoids found in licorice, as there is the potentiality to provide natural interventions against COVID-19 symptoms.

## 1. Introduction

The coronavirus disease (COVID-19), which began in the city of Wuhan in the Peoples’ Republic of China (PRC) during the last quarter of 2019, quickly moved to Europe and the USA and subsequently into the Asian and African countries. This severe acute respiratory syndrome coronavirus-2 (SARS-CoV-2) has now infected millions of people worldwide and has taken more than 5 million lives as of the end of October 2021 [1]. At the time of writing (October 2021), no pharmaceutical intervention was developed to treat COVID-19 symptoms, specifically ARDS, but vaccination has been quickly going on in various parts of the world. Although most countries are providing vaccines to their citizens as quickly as possible, hundreds of thousands of breakthrough cases are reported from all around the world, specifically from India and Israel [2,3,4,5,6,7,8,9,10]. As it may take several months or years to vaccinate all the people worldwide, many countries still follow curfews and lockdown measures, which clearly affect personal freedoms and impinge heavily on economic progress. Even after a year of starting, tens of thousands of business offices have been closed, with staff working from their home environment. Apart from this, hundreds of thousands of schools, colleges, and universities across the world have been closed, with students forced to learn online. As a result of these disruptions, many people struggle to find income to support their families, and several national and state governments have been required to ensure sufficient revenue flow within their communities. This situation will only be relieved with the development and availability of symptomatic control medicines or by vaccinating the whole world against all the existing and potential strains. The presence of hundreds of thousands of breakthrough cases highlights the significance of pharmacological interventions to treat COVID-19 cases.

The severe symptoms which affect individuals suffering from COVID-19 have been termed ‘Acute respiratory distress syndrome (ARDS)’ and include severe inflammation of the lungs, blood clots, and multi-organ failures [11]. Studies have also found lymphopenia with T-cell hyperactivation, leukopenia, and thrombocytopenia among 40% of the affected patients [12]. It has been claimed that the formation of cytokine storms in the body and depositions in the lung alveoli related to the viral clearance mechanisms is considered responsible for the severity of the condition and the need for hospitalization [12,13]. Further, the presence of ferritin and interleukin–6 (IL-6) are considered to be potential biomarkers in predicting mortality, strengthening the conviction that hyper inflammation is responsible for patient hospitalization, which often requires intensive ventilation [12]. It would thus appear that if any pharmaceutical intervention can prevent viral replication in the body (antiviral property) and inhibit severe lung inflammation (anti-inflammatory property), many lives can be saved from COVID-19 infection.

It is well known that inflammation is a physio-pathological condition characterized by the body’s complex biological response to physical, chemical, and biological stimuli. It is an adaptive response, which is triggered by injuries or pathogen invasions in the body. In the modern medical system, several antiviral and anti-inflammatory drugs are generally useful. These include hydroxychloroquine, chloroquine, ivermectin, tocilizumab, adalimumab, and ruxolitinib, all of which have been tried against the COVID-19 symptoms, with little curative effects [14]. Later studies questioned the efficacy [15] and none of them are found completely successful [16], which leaves the current COVID-19 treatment system as supportive care.

In the context of this state of uncertainty and lack of appropriate pharmacological interventions, this review article suggests looking into the plant kingdom to develop an appropriate drug to treat COVID-19 patients. It has been suggested after observing the beneficiary effects of several plant species in treating a number of significant diseases. For example, an alkaloid from the bark of the Cinchona tree (*Cinchona officinalis*; locally known as Quina Quina, meaning bark of bark or holy bark) named quinine was used with good effect to treat malaria [17]. The same quinine leads to the development of chloroquine and hydroxychloroquine, which were mentioned as modern antiviral and anti-inflammatory drugs. (The late Winston Churchill once said that ‘Drinking gin and tonic (from the Cinchona tree) has saved more English men’s lives and minds than all the doctors in the Empire’, which highlights the significance of the Cinchona plant in mitigating and eradicating malaria.) In addition to Cinchona, the identification of the Colchicum plant (*Autumn crocus*) and its use in treating gout and Mediterranean fever [18] was a significant breakthrough in medical history. Other medicinal plant species have also been identified and assessed as having antimicrobial (including antiviral) or anti-inflammatory properties or, indeed, having both at the same time [19,20,21]. In India, the major traditional treatment system known as ‘Ayurveda’ (meaning ‘longevity’) has been used in phytochemicals found in hundreds of identified plant species to treat several kinds of diseases for 5000–6000 years [22,23]. Similarly, traditional Chinese medicines use medicinal plants in their treatment protocols [24,25]. During the last two decades, several traditionally used medicinal plants have been subjected to study, and many alkaloids and flavonoids have been identified, which can treat several diseases [26].

In the absence of any suitable pharmaceutical intervention in western medicine to treat COVID-19 patients, the objective of this review is to highlight the potential of a medicinal plant species, commonly called licorice (*Glycyrrhiza glabra*), which belongs to the shrub category, whose phytochemicals have antiviral and anti-inflammatory properties, and to suggest that clinical trials be introduced to assess its potency regarding COVID-19 symptoms. Phytochemicals from many other medicinal plants are also known to have similar properties, such as *Sambucus nigarac* [27], *Desmodium canadense*, *Lamiaceae* family, *Asteraceae*, *Geraniaceae*, etc. [28] but licorice has explicitly been suggested due to its: (i) considerable antiviral property against several viruses, including SARS-CoV, (ii) strong anti-inflammatory property, which has been observed in many rat model studies, (iii) autophagy-enhancing mechanism, (iv) established use in Chinese and Indian Ayurvedic medicines, and (v) wide distribution.

## 2. Methodology

Searches were conducted in the existing literature through Google Scholar, PubMed, Scopus, and Web of Science to obtain the most up-to-date research information regarding licorice’s antiviral and anti-inflammatory properties. More broadly developed articles have not been included since this article’s objective is to focus on licorice’s antiviral and anti-inflammatory properties plus its autophagy-enhancing mechanism. To assist the discussion, this review contains a brief description of the mechanisms of inflammation in the human body and the actions of phytochemicals on it. Apart from general information about licorice, its antimicrobial, specifically antiviral, and anti-inflammatory properties, together with its autophagy-enhancing mechanism, are included. The review ends with a small discussion and conclusion.

## 3. Phytochemical Actions on the Human Body

Disease treatment with medicinal plants is common in many ancient cultures, including Indian, Egyptian, African, Chinese, Japanese, American Indian, and Australian Aboriginal [29,30,31]. This practice has a history of more than 5000 years, and it is still a significant source of health care for large numbers of people, particularly in the developing areas of the world [32]. The World Health Organization (WHO) has indicated its understanding of the significance of medicinal plants’ treatment and has listed more than 21,000 medicinal plants [33]. Medicinal plants are used in the Ayurvedic treatment system for obesity, fatigue, multiple sclerosis, cardiovascular disease, asthma, depression, arthritis, cerebral injury, lung fibrosis, and many other diseases. Metabolic diseases, including infection and inflammation, are also treated in this fashion [34]. In addition to their use in the Indian subcontinent, herbal medicines are commonly used in China for medical treatment. Even though many respected cultures have used medicinal plants for thousands of years, only very recently have pharmacologists tried to identify and assess the healing potential of the phytochemicals inherent in these medicinal plants. In addition, herbal therapeutic effects on mitochondrial functions have been studied by many researchers [35,36,37].

**Figure 1:**
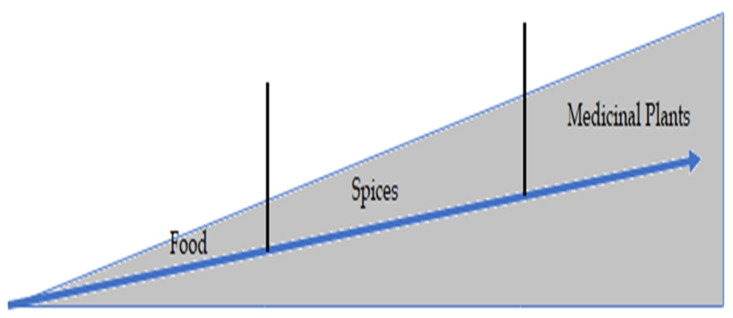
Conceptual diagram showing the comparative influence (on Y-axis) of food, spices, and medicinal plants (on X-axis) on the human body [38].

When food is ingested, it can be either used for normal body functioning and growth, or in some circumstances, for healing purposes. Experience has shown that spices generally have an enhanced healing effect, but in the case of specific medicinal plants, the healing effect is much more significant. This has been summarized in a conceptual diagram showing the effect of food, spices, and plant-based medicines in Figure 1. Of particular interest here is that when a foreign material, for example, a modern drug, comes inside the body, the body can recognize it as foreign material and may reject it. The body may send it to the liver and come out through the urine, termed ‘First pass effect.’ In contrast, when a plant-based medicine is ingested, it is more likely to be accepted without stress because the body recognizes it as natural, like vegetables and fruits that we eat daily [37].

It is relevant to note that plants do not have a comparable immune system to animals to act against microbial pathogens; instead, they work through a defense mechanism that induces various types of antimicrobial compounds such as proteins, peptides, and small molecular weight organic substances. These compounds are found to have significant therapeutic effects in animals and humans [20]. Consistent with this observation, through various recent studies, it has been found that many medicinal plants used for traditional treatments have their own phytochemicals suitable for extraction to provide therapeutic and healing effects [39,40]. These phytochemicals include alkaloids, flavonoids, terpenoids, and carotenoids, including nitrogen-containing compounds, with many having antimicrobial and anti-inflammatory properties [39,40,41].

In the literature, around 100 British Colombian medicinal plants have been identified, some with antiviral properties, including more than 10 with significant antiviral activity against the respiratory synclinal virus (RSV), herpes virus-1 (HSV-1), coronavirus (CoV), and parainfluenza virus-3 (PI3) [42]. Similarly, more than 800 Chinese medicinal plants were identified to have antiviral properties [43], including some useful against SARS-CoV [44]. It is also recognized that some of the plant extracts have shown efficacy against existing conventional drug-resistant viruses [45,46]. Several researchers studied the inhibitory effects of plant-based biomolecules on various pathogenic viruses such as Human immunodeficiency virus (HIV) [39,47], Hepatitis B virus [48], Herpes simplex virus–type 2 [49], and SARS-CoV [41,50] with successful results. Essential oils from several tree species have also shown antimicrobial, and specifically, antiviral properties. For example, it is reported that essential oils from Tea trees, Eucalyptus trees, Citrus spp., and *Hyssopus officinalis* could alter the fluidity of the viral membrane by insertion into the lipid double layer envelope, generating an inhibitory effect [21,51,52,53,54,55]. These known examples suggest that there may be many more potential antimicrobial compounds, specifically antiviral and anti-inflammatory agents, that need to be explored in the form of phytochemicals. Therefore, this review explores both the antiviral and anti-inflammatory actions and the autophagy-enhancing mechanism of licorice to suggest clinical trials for COVID-19 symptoms.

## 4. Licorice (*Glycyrrhiza glabra*)

**Figure 2:**
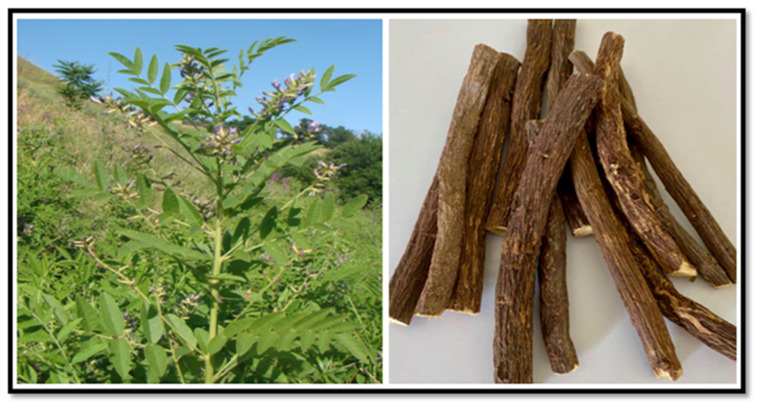
Licorice plant (**left**) and its root (**right**).

Licorice (also called liquorice) is a Greek word meaning ‘sweet root’ and is a perennial herb native to southwestern Asia and the Mediterranean region in Europe [56] (Figure 2). The plant has been used in several locations in India, China, Greece, Europe, the Middle East, and Africa for various treatments, particularly those related to arthritis and ulcers [57]. It is known as ‘Yashtimadhu’ (sweet root) in Sanskrit and ‘Gan cao’ (sweet grass) in the Chinese language [57] and was also used in Arabic medicine in the Middle Ages and documented in the Canon of Ibn Sina (980–1037 AD) [56]. The herb belongs to the *Glycyrrhiza glabra* species in the Leguminosae family and grows to a height of around 2 m [58].

The plant has a long cylindrical-shaped, multi-branched root, which extends horizontally underground and is mainly used for medicinal purposes [59]. Licorice is 50 times sweeter than sugar due to the presence of glycyrrhizin (glycyrrhizic acid-GL) and is often used as a sugar substitute [59,60]. The plant has antioxidant properties; therefore, it is used in some cancer treatments [59,61]. The licorice family has three original plants used for treatment: *G. uralensis, G. inflata,* and *G. glabra* [62]. The licorice plant is considered a weed in many places like wheat crops and cotton plantations, together with potato, sugar beet, clover, and sainfoin fields, but it has been used as traditional medicine in many ancient civilizations such as for the Greeks, Romans, Egyptians, Assyrians, Indians, and Chinese [55,63].

Licorice falls into the list of first-line medicinal plants [64,65,66]. In ancient China and India, licorice has been used for more than 5000 years to treat respiratory and liver disease and alleviate the toxicity of other drugs [56,62,63]. The Ayurvedic Pharmacopoeia of India [63] mentions that licorice treats inflammation, eye and liver diseases, throat infections, peptic ulcers, and arthritis. Greeks used licorice to treat both gastric and peptic ulcers, whilst Europeans and Asians have utilized it to treat psoriasis [67]. It is a traditional Persian medicine to treat various diseases, including respiratory disease [68]. The role of licorice in Japan also needs to be highlighted as they have been using it to treat chronic hepatitis [68]. The plant also has anticancer, hepatoprotective, antispasmodic, neuroprotective, antioxidant, and estrogenic properties and is very useful in reducing hepatocellular damage in chronic hepatitis B and C patients [69]. For many years, it has had the reputation of being a memory booster [70] and an antidepressant [71] and can reduce blood cholesterol levels [72,73] and acts as a promising drug for treating liver and renal complications [74,75,76]. It is also found that the plant can reduce polydipsia and frequent urination in diabetic patients [69]. In some places, the root is used to prepare tea, and the dried root is used as a tooth cleanser [77].

When studies on the properties and therapeutic benefits of phytochemicals in the licorice have been undertaken, scientists discovered that they could extract more than 20 triterpenes, 300 flavonoids, and 73 bioactive compounds from the root and identified 91 potential targets for its action [62,78,79]. Many of the extracted bioactive compounds were shown to have antimicrobial, antiviral, and anti-inflammatory properties, such as GL, 18ἁ/β-Glycerrhetinic acid (GA), three triterpenes, and several flavonoids (Table 1).

The roots contain several phytochemicals such as 2β-GL, glucuronic acid, GA, tannic acid asparagine, resins, volatile oils, flavonoids such as liquiritigenin (LG), liquiritin (LQ), isoliquiritigenin, isoliquiritin, and coumarin compounds such as herniarin and umbelliferone. They also contain glabridin compounds such as glycerin flavone, glabrene, glabryl, formononetin, and isoliquiritigenin [68]. Many of these compounds have several known benefits and have been used as neuroprotective, antidepressive, oestrogenic, sedative, antimicrobial, specifically antiviral, anticarcinogenic, immunoregulatory, hepatoprotective, and antioxidant properties [62,94].

### 4.1. Antimicrobial Activity

Many studies revealed the antibacterial activity of the plant (various extracts and flavonoids) against several bacteria strains, including *Helicobacter pylori* and methicillin-resistant *Staphylococcus aureus* (MRSA) [58,84,92,95,96,97]. Mass and Cock [91] studied the antibacterial efficacy of Licorice and demonstrated that the acetate root extract exhibited strong antibacterial efficacy against *K. pneumonia* and *A. baylyi*, and Wu et al. [92] found that the flavonoids Glabrol, Licochalcone A, C, and E are very effective against *Staphylococcus aureus*. The activity of GA against MRSA was studied by Long et al. [84] and they found that the acid inhibits MRSA survival and attenuates virulent gene expression. Later, Celik and Duran [83] found that the same GA is very effective against *Helicobacter pylori*. Zhou et al. [98] believe that licochalcone could be used to synthesize novel anti-*S. aureus* compounds that may inhibit the production of ἁ-toxin in methicillin-sensitive *S. aureus* (MSSA) and MRSA. In addition to antibacterial efficacy, the antifungal efficacy of licochalcone and glabridin has been observed by Messier and Grenier [90].

#### 4.1.1. Antiviral Activity

##### In Vitro Studies

**Figure 3:**
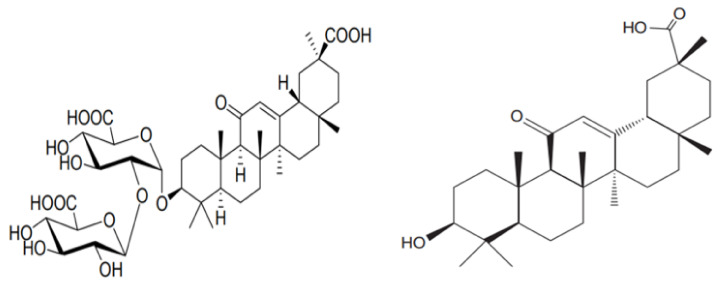
Chemical structure of Glycyrrhizic Acid (GL) (**left**) and 18β-Glycerrhitinic acid (GA) (**right**).

One of the significant characteristics of licorice, which may be useful for treating COVID-19, is its antimicrobial property, particularly its antiviral effect [95,99]. The antiviral property of licorice is revealed by many researchers, with the first study being published in 1979 [100], whose finding was that GL and GA (structure in Figure 3) are the main compounds behind the antiviral efficacy [101]. Ashfaq et al. [102] investigated the antiviral characteristic of licorice against the hepatitis C virus. They demonstrated that the plant extract could inhibit hepatitis C virus’ growth, including a 50% reduction (14 ± 2 µg/mL) in viral concentration (including assessment of the full-length particle and core gene expression). Through the experiment, Matsumoto et al. [101] found that GL targets the release step of the hepatitis C viral infection, which identified a potential role for GL in hepatitis C treatment. Huang et al. [103] studied the effect of GL on HIV infection and found that GL perfusion can inhibit HIV infection by reducing its adhesion and stress components. Later, the efficacy of GL against Coxsackievirus A16 (CVA16) and Enterovirus 71 (EV71) was studied by Wang et al. [57]. GL’s effectiveness against the influenza virus was also studied, and it was observed that GL could inhibit the H5N1-induced production of chemokine ligand 5 (C-C motif CCL5) and ligand 10 (C-X-C motif, CXCL10), together with IL-6 [104,105,106]. It was also found that GL can suppress H5N1-induced apoptosis activity at a concentration of 100 µg/mL [104]. Apart from this, the action of GL against the herpes simplex virus (HSV1) is studied by Laconi et al. [107] and found that pre-treatment with GL on the HeLa cell improved the antiviral property (when experimented with HSV1) by a factor of 95 to 98%. Besides viral inhibition, GL also evidences immunostimulant activity against viruses such as the duck hepatitis virus (DHV) [108]. Both antiviral and antitumor activity of licorice root extracts were investigated by Fukuchi et al. [109], who found that the alkaline extracts demonstrated higher antiviral activity against HIV compared to the water extract. This led the authors to suggest that this extract may be converted into mass production as an anti-HIV agent.

An epidemic with a severe coronavirus (SARS-CoV) started in 2002 in China and spread to 32 countries. After this epidemic, many researchers studied the efficacy of licorice, specifically GL, on SARS-CoV. The study of Hoever et al. [110] is significant, and their in vitro study revealed that GL was able to inhibit virus replication. Among the 15 GL derivatives, 2-acetamido-β-D glucopyranosylamine, when inserted into the glycoside chain of GL, showed a ten-fold higher antiviral efficacy than normal GL. It was also observed that GL could inhibit the absorption and penetration of the SARS-CoV in the early replicative cycle, specifically when given during and after the absorption period. However, due to the complexity of this mechanism, the exact activity details are unclear, but there is a suggestion that nitrous oxide (NO) donation is somehow involved [56,111].

Apart from GL, the antiviral activity of GA was also studied against many viruses, including the human respiratory syncytial virus (HRSV), arbovirus, vaccinia, and vesicular stomatitis. It was identified that both GL and GA could induce interferons that can bind to cell surfaces and stimulate the synthesis of intracellular proteins, blocking the transcription of viral DNA [112]. Interferon also activates the macrophages and stimulates the augmentation of the natural killer cell activity [112]. All of these studies demonstrate that GL and GA in licorice, particularly GL, can be used as a potential antiviral drug (Table 2). Therefore, it is suggested to undertake in vitro and in vivo studies followed by clinical trials to investigate the antiviral efficiency of GL against SARS-CoV-2 replication.

##### In Vivo Studies

The effect of GL on the influenza virus was conducted in a mouse model study more than two decades ago. The mice were treated with 10 mg of GL/kg body weight intraperitoneally (IP) one day before exposure to the virus (lethal dose able to kill around 50% of the animals). The result was successful, with all the GL-treated animals surviving the experimental period of 21 days, whereas, for the control animals, the mean survival time was only 10.5 days [127]. The efficacy study of GL in murine herpes encephalitis revealed that the IP administration of GL increased the survival rate of animals by 2.5 times, and the viral replication in the brain was found to reduce more than 45% [128]. A study with Coxsackievirus B3 (CVB3) revealed that GL is a factor in improving the state of Coxsackievirus B3 (CVB3)-induced myocarditis [124].

##### Human Studies

During the SARS-CoV epidemic in China (2002–2003), Lu et al. [129] conducted a clinical trial with GL against SARS-CoV on the virus confirmed patients. Among the 73 patients, 37 were treated with GL. After the complete treatment regimen, the major symptoms vanished quickly in the treated group compared to the placebo. Another trial was conducted on 60 SARS-CoV patients, with half belonging to the interventional group. The average period from peak severity of the lesions to 50% improvement was shorter in the interventional group treated with GL [130].

### 4.2. Anti-Inflammatory Property

Inflammation plays a significant role in epidemic and pandemic diseases, and licorice is considered an alternative choice for the treatment [62,131]. Inflammation is primarily a protective measure against microbial invasion, which includes action against the presence of toxins or allergens. However, in some cases, such as COVID-19, it may become uncontrollable and detrimental to the tissues and organs [132,133]. It is the primary cause of many human diseases such as asthma, rheumatoid arthritis, and atherosclerosis [134]. The inflammatory response occurs as a result of the production of pro-inflammatory cytokines such as IL-1, IL-6, IL-12, IL-18, interferon (INF)-γ, tumor necrosis factor (TNF), and granulocyte-macrophage colony-stimulating factor [134,135]. The activity of the nuclear factor-kB (NF-kB) and transcription factors also play a significant role in inflammation by regulating the expression of various genes that encode the pro-inflammatory cytokines, adhesion molecules, chemokines, growth factors, and inducible enzymes such as cyclooxygenase-2 (COX-2) [135].

Since ancient times, licorice has alleviated pain, relieving coughing, eliminated phlegm, and treated respiratory, liver, and gastric diseases [136]. Like its antimicrobial activity, the anti-inflammatory activity (microbial induced inflammation) of licorice and its mechanism has been studied by many researchers [59,62,137,138,139]. It has been found that the action is similar to those of glucocorticoids and mineralocorticoids and is mainly due to the presence of GL [139,140,141] and GA in Licorice [59,141]. Glabridin, liquiritin, liquiritigenin, and licochalcone, including 13 flavonoids, are present in licorice, in addition to GL and GA, and all these compounds have shown significant anti-inflammatory activity [62,86,142]. Therefore, these compounds are used against liver and renal complications [143]. The following are some of the studies that highlight GL and GA’s influence in treating various inflammatory diseases.

#### 4.2.1. In Vitro Studies

There are several in vitro studies conducted to investigate the anti-inflammatory property of licorice, specifically the effects of GL and GA. Wang et al. [144] investigated the same effects in lipopolysaccharide (LPS) stimulated macrophage model on RAW264.7 cells by treating with 25–75 µM GA or 18βGA and found that both are potential agents for the treatment of inflammatory-mediated diseases. They realized that both compounds inhibited the NF-kB activation and the activities of phosphoinositide-3-kinase (P13K) and reduced the production of LPS-induced tumor necrosis factor-ἁ (TNF-ἁ), IL-6, and IL-1β in a dose-dependent manner [144]. Similarly, Bai et al. [145] explored the anti-inflammatory effect of licorice residues and reported that a compound in the residue (compound 18) displayed the highest anti-inflammatory effect (No inhibitory effect) in the RAW264.7 cells. Further studies revealed that the anti-inflammatory effect happened through the downregulation of the pro-inflammatory cytokines (IL-1β, IL-6, inducible nitric oxide synthase (iNOS), and cyclooxygenase-2 (COX-2)) [145]. Apart from GL and GA, one licorice extract named licoflavanone also showed strong anti-inflammatory activity in LPS-stimulated RAW 264.7 murine macrophages [146].

#### 4.2.2. Animal Studies

Aly et al. [59] studied the anti-inflammatory activity of licorice using the carrageenan-induced edema model in male albino rats at the Al-Isra University in Jordan. They found that aqueous licorice extract and GA in licorice demonstrated significant anti-inflammatory activity similar to diclofenac sodium (DS). Similarly, in a mouse model study, Xiao et al. [147] investigated the influence of GA in *Propionibacterium* acnes-induced acute inflammatory liver injury. They found that GA exhibits anti-inflammatory effects through the inhibition of pro-inflammatory cytokines (such as IFN-γ and TNF-ἁ), P. acnes-induced NF-kB activation, and chemokine expression (MIP-1ἁ). Another investigation on the anti-inflammatory effects of GL found that it significantly inhibited the LPS-induced inflammatory response in a mouse by inhibiting the TLr4 signaling pathway [148].

The anti-inflammatory activity of GA and hydroxypropyl γcyclodextrine was investigated against small intestine injury on indomethacin-treated mice. A significantly high plasma concentration of GA was detected after the oral administration of the compound [149]. It was also found that 18β-glycyrrhetinic acid-hydroxypropyl-γcyclodextrin compound reduced the mRNA expression of the IL-6, IL-1β, including TNF-ἁ and thus showed a potential therapeutic value against indomethacin-induced small intestine injury [149]. The ethanol extract of roasted licorice was also reduced in the TNF-ἁ and IL-6 and increased IL-10 in LPS treated mice, which facilitated the survival rate [150]. Apart from this, many other studies also showed the anti-inflammatory property of licorice on live animals [151], and the details are summarized in Table 3.

It was reported that IP administration of GL suppressed the lung inflammation caused by the infection of *Streptococcus aureus* in a mouse model study [152]. Further to this, it has been mentioned that GL has a protective effect against TLR4 activator LPS-induced acute respiratory distress syndrome (ARDS) in mice [153]. Similarly, in a mouse model experiment, it is observed that GL reduced the mortality of influenza-infected mice by interferon γ and T cell activation [127]. Menegazzi et al. [154] injected carrageenan (a well-known acute model inflammation widely used for inflammatory research) into the pleural cavity of mice to investigate GL’s influence in reducing inflammation, and they observed that the injection resulted in inflammation with fluid accumulation in the pleural cavity, mainly as a result of the production and accumulation of TNF-ἁ and IL-1β. The researchers noted that these inflammation events occurred as a result of the activation of NF-kB, including the activation of signal transducer and activator transcription-3 (STAT-3) in the lung [154]. However, surprisingly GL inhibits these activation results in the reduction of inflammatory response.

**Table 3:** The anti-inflammatory property of licorice extracts in various studies.

Compound	Tissue/Disease	Concentration	Method	Inhibition Rate	Reference(s)
**In vitro studies**					
8β-GL	LPS (1 μg mL^−1^)-induced l Murine cell (RAW 264.7)	75 μM	ELISA	51%-NO, 51%-IL-1β, 49%-PGE2 & 42%-IL-6	[144]
18β-GL	LPS (1 μg mL^−1^)-induced murine Cell (RAW 264.7 macrophages)	0.5 or 1 mg mL^−1^	ELISA	Supress PGE2, PGI2,TXB2 & LTB4	[155]
18β-GL	*Leishmania donovani* infected Macrophages-BALB/c mice (age: 4–6 weeks)	50 mg mL^−1^	ELISA	90.94%-parasite load	[156]
18β-GA	LPS (1 μg mL^−1^)-induced murine cell (RAW 264.7 cell)	75 μM	ELISA	34%-NO	[144]
18β-GA		75 μM	ELISA	58%-PEG2, 42%-1L-1β, 35%-IL-6, 34%-TNF-ἁ	[144]
LID	LPS (0.1 μg mL^−1^)-induced U937 Cell line (human monoblastic leukaemia cell line)	0.1, 0.5, 1 μg mL^−1^		Decreased the secretion of IL-6, MMP-7, MMP-8, & MMP-9	[157]
DGC	Glutamte (5 nM)-induced HT22 cells	2 μM	2,7-DCF assay	Dose-dependent inhibition of ROS assay & WB production	[158]
LIA	LPS (0.1 μg mL^−1^)-induced U937 cell line (human monoblastic leukaemia cell line)	0.1, 0.5, 1 μg mL^−1^		Decreased the secretions of CCL5 @ (1 μg mL^−1^), MMP-7 @ (0.5, 1 μg mL^−1^) MMP-8 @ (0.5, 0.1, 1 μg mL^−1^)	[157]
LCA	LPS (μg mL^−1^) induced murine cells (RAW 264.7)	3 &10 μM	DCFH-DA	>80% PGE2 inhibition @ 10 μM fluorometric >50% NO inhibition at	[151]
18β-GL	LPS (μg mL^−1^) induced	75 μm	ELISA	51% reduction in NO	[144]
	Murine cells (RAW 264.7 cells)			51% reduction in IL-1β 49% reduction in PGE2 46% reduction in TNF-ἁ 42% reduction in IL-6	
18β-GA		75 μm	ELISA	58% reduction in PEG2	[159]
Glabridin & isoliquiritigenin		20-40 μg mL^−1^	Cell culture & cell viability assay	anti-inflammatory activity is due to the individual or synergistic effects	
**In vivo studies**					
**Compounds**	**Inflammation Details**	**Models**	**Treatment**	**Result(s)**	**Reference**
18ἁ-GL	20% paraquat poisoning solution @ 15 mg kg^−1^	Sprague Dawley rats-male 30 Ns (180–200 g)	injection-IP 30 mg kg^−1^	Significant decrease in intercellular adhesion molecules (ICAM-1) and matrix metalloproteinase-9 (MMP-9)	[147]
18β-GL	LPS (1 mg kg^−1^)-Intratracheal installation	BALB/C mice (male 20–25 gm)	injection-IP 10, 25 & 50 mg kg^−1^	Noted decrease in NO and MPO activity	[160]
LCA	Topical inflammation induced instantly at the posterior surface of the ear (using xylene 0.05 mL)	Kunming mice (20–25 gm) & Wistar rats (150–200 gm)	50 mg kg^−1^	Decrease in ear oedema rate by 30.3%	[161]
**Human Studies**					
GL	Hepatitis B virus induced inflammation	Humans	oral and IV (60 mL daily) for a week)	Effective in normalizing serum (for 7 days, later 3 days transaminases)	[115]
GL	Hepatitis C virus induced inflammation	Humans	40 mL transaminases	Found effective in normalizing serum	[116]
GL	Hepatitis virus induced Inflammation	Humans	40 mg of GL (IV)	Suppressed ALT	[80]

NB: GL—Glycyrrhizin; GA—glycyrrhetinic acid; LID—licoricidin; DGC—dehydroglyasperin; LIA—licorisoflavan A; LCA—licochalcone A; LCB—licochalcone B; LCC—licochalcone C, LCD—licochalcone D; LCD—licochalcone D; LCE—licochalcone E; ALT—alanine aminotransferase; IP—Intraperitoneal.

#### 4.2.3. Human Studies

The influence of licorice extracts, GL and GA, on humans was studied by many researchers, who found that both effectively inhibit the viral replication and inflammatory response [81,114,162]. Miyake et al. [80] conducted a study that administered 40 mg of GL by injection to patients with chronic viral hepatitis and evaluated dose-response levels, including the frequency of administration. They found that GL effectively suppressed the alanine aminotransferase (ALT) in patients [80]. Zhang and Wang [82] also mentioned the efficacy of GC in lowering the ALT levels in chronic hepatitis B patients. In the LPS model of inflammation, GL can reduce TLR4 expression in the lung and the heart by significantly reducing the cytokine release [153,163].

#### 4.2.4. Mechanism

The mechanism behind the anti-inflammatory activity of GL and GA is very complex. Antimicrobial activity is considered one of the best anti-inflammatory methods as microbial inhibition reduces inflammation [141]. It is reported that GL makes the anti-inflammatory activity by influencing the adrenal gland and thereby stimulating the body’s own anti-inflammatory adrenal steroid hormone, named cortisol, whenever required. It can also break down the post-action of the excess cortisol [137,164]. Studies reported that GL and GA could activate cortisone activity by (i) binding with glucocorticoid receptor (GR) signaling need for production [165], (ii) inhibiting the activity of corticosteroid 11β-dehydrogenase isozyme 2 (11β-HSD2), which usually converts active cortisol into inactive cortisone [166], and (iii) eliminating the oxidative stress within the body [167].

**Figure 4:**
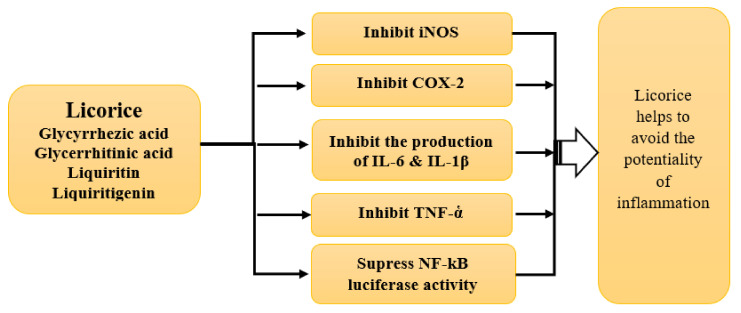
Schematic diagram showing the action of licorice in inhibiting the inflammation.

The high-mobility group box 1 protein (HMGB1) is a nuclear component but acts as a signaling molecule in acute and chronic inflammation [168]. Anti-inflammatory action is characterized mainly by the promotion of immunity, inhibiting the pathogen and pathogen-induced macrophage responses, specifically by binding with high-mobility group box-1 (HMGB1) and modulating P13K signaling [142,167,168,169,170]. According to Yu et al. [142], inducible nitric oxide synthase (iNOS) and cyclooxygenase-2 (COX-2) are responsible for the over-production of cytokines and inflammation. Therefore, inhibition of iNOS and COX-2 is considered the most efficient approach in inhibiting the inflammatory response and the subsequent disorders. After a detailed study, Yu et al. [142] demonstrated that active licorice extract components GA, LQ, and LG could strongly (i) inhibit the production of NO in mice microglial cells (LPS activated), (ii) suppress the expression of IL-6, IL-1β, TNF-ἁ in LPS treated cells, and (iii) attenuate the COX-2 and iNOS expressions in LPS stimulated BV2 cells (Figure 4). Bodet et al. [169] also investigated the anti-inflammatory efficacy of licorice on periodontal disease using a super-critical carbon dioxide extract. They found that the extract exhibits anti-inflammatory activity by inhibiting the periodontal-pathogen-induced macrophage responses (IL-6, IL-8, IL-1β, and TNF-ἁ) and phosphorylation of macrophage intracellular signaling proteins. The licorice extract also inhibited the pro-inflammatory cytokine response in the ex vivo human whole blood model [169]. Studies in rheumatoid arthritis and periodontitis patients revealed that TNF-ἁ is an autocrine stimulator and a potent paracrine inducer of pro-inflammatory mediators, including interleukins (IL-1, IL-6, and IL-8) and is a granulocyte-macrophage colony-stimulating factor [171,172]. When a licorice extract was applied to a HaCaT human keratinocyte cell line, it attenuated the tumor necrosis factor-ἁ (TNF-ἁ) and chemokine production by the interferon-γ mediated pathway by targeting the STAT-1 and NF-kB signaling (in keratinocyte) pathways [173]. It also has been shown to control the production of PGE2 by the synovial cells that cause tissue destruction [174].

Because of its anti-inflammatory property, both GL and GA can protect rat hepatocytes from bile acid-induced cytotoxicity [175]. The beneficial effect of GL on hepatitis patients was observed when intravenously administered, resulting in decreased serum ALT, necro-inflammation, and liver fibrosis [176]. Due to its strong anti-inflammatory activity, Li et al. [177] and Ye et al. [178] suggested that licorice could be used to treat renal and liver problems. The study of Zhang et al. [179] is also significant when considering the case of COVID-19, as their research demonstrated the inhibition of pro-inflammatory cytokines (nuclear factor-kB, interleukin-1β, interleukin-6) in CVB-3-induced myocarditis patients. In addition to the above studies, randomized controlled trials revealed that GL and its derivatives could reduce hepatocellular damage caused during chronic hepatitis B and C [180]. Most of the studies used licorice extract, but a 2010 study highlighted that roasted licorice has more potent anti-inflammatory activity compared to raw licorice [181].

In the pulmonary system, NF-kB activation is considered a focal pathway in generating inflammation, and several noxious stimuli are responsible for this activation [182]. However, through rodent model studies, it has been observed that pre-treatment with nonspecific inhibitors can reduce inflammation [183]. The angiotensin-converting enzyme -2 (ACE-2) is the main pathway in which the SARS-CoV-2 enters the host cell. Therefore, any actions that reduce the number of ACE-2 are considered as safe. However, from the inflammation point of view, it has been observed that ACE-2 suppresses the toll-like receptor 4 (TLR4) and hence the inflammation in the lung. Therefore, the reduction in ACE-2 expression is controversial in nature. In addition to GL and GA, licochalcone has also shown significant anti-inflammatory activity (in vitro and in vivo) by suppressing the NF-kB activation and p38/ERK MAPK signaling [184]. These studies (Table 3) confirmed the high potential for GL and GA to act as a novel therapeutic intervention to alleviate inflammation, specifically in COVID-19 patients.

### 4.3. Effect on Autophagy

Autophagy is a cellular mechanism that cells use to adapt to stress conditions, including the internal invasion of pathogens. The process helps to clear out the pathogens, thereby reducing pathogen replication and the subsequent inflammatory consequences. However, some viruses like SARS-CoV-2 and HSV-1 can inhibit autophagy mechanisms to suit their replication conditions. This mechanism is usually linked to the inhibition of Beclin-1, which is considered one of the first proteins that a cell produces in the autophagic process [185,186]. In addition to its antiviral and anti-inflammatory properties, it is found that the GL can induce the autophagy mechanism in the cells by increasing the concentration of Beclin-1 [107]. After 24 h of treatment with GL, it was observed that the Beclin-1 production significantly increased (*p* < 0.01) (by two-fold to three-fold) in comparison with rapamycin treatment (Figure 1 in [107]). This is an important requirement in SARS-CoV-2 infected cells, as the SARS-CoV-2 virus inhibits Beclin-1 levels by inducing the production of SKP2 proteins [187,188]. When GL was added to the cell 24 h before adding the herpes simplex virus (HSV1), Becklin production increased, and the cell demonstrated higher antiviral effects. The pre-treatment (2mM) increased the Beclin-1 production around five-fold compared to that induced at time zero. When GL was added to the HeLa cell together with HSV1, a strong antiviral activity was exhibited, whereas by comparison, the more traditional rapamycin treatment did not show any activity [107]. This finding highlights GL’s ability to act as a prophylactic against HSV1, and it is suggested that it may also work against other viruses, including SARS-CoV-2.

## 5. Discussion

Based on various studies conducted across the world, it is clear that licorice has potent antimicrobial, anti-inflammatory, anticancer, and antioxidant properties [74,189,190]. Studies revealed that GL is hydrolyzed to enoxolone (GA) by intestinal bacteria in the gut. After absorption from the gut, the same GA is metabolized into 3β-monoglucuronyl-18β-glycyrrhetinic acid in the liver [74]. The resulting metabolite is able to circulate in the liver, and hence the oral bioavailability is poor compared to intravenous administration [191]. To support this, through a rat model study, Egashira et al. [192] also reported that the IV and IP (intraperitoneal) administration of GL could provide more bioavailability than oral bioavailability. Among the 20 triterpenoids and more than 300 flavonoids present in licorice, two particular triterpenes, GL and GA, are thought to be mainly providing antimicrobial activity. In addition to GL and GA, the flavonoid named chalcone has a significant role in inhibiting bacterial infections. Analysis of the licorice plant has indicated that its underground portion is the most bioactive part [193]. Multiple studies have established that GL and GA organize viral inhibition through three specific activities: (i) inhibiting the gene expression and replication of the virus, (ii) minimizing adhesion force and facilitating stress reduction, and (iii) reducing the DNA binding ability of HMGB1 [141,142,146,173]. In contrast, licochalcones inhibit bacterial infection by (i) bacterial gene reduction, (ii) bacterial growth inhibition, and (iii) reduction of toxin production [92,194]. A dose-dependent therapeutic response was also observed in hepatitis patients [80], which is understood to suppress the alanine aminotransferase (ALT) levels in patients with chronic symptoms. It has also been found that GL can prevent tissue injury caused by chronic hepatitis and many other diseases [180]. Of particular interest to this discussion is that the significance of GA in treating inflammation was observed by Shi et al. [195] in a mouse model study. They found that treatment with GA inhibited hepatic inflammatory activity by blocking the high mobility group box-1 (HMGB-1) cytokine activity, suggesting a new therapy for acute viral hepatitis. In addition to this finding, a second mouse model study highlighted the significance of GL in protecting mice exposed to the influenza virus [127].

**Figure 5:**
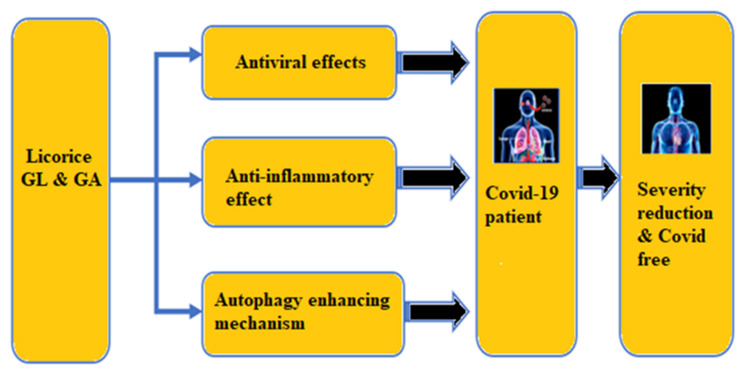
A conceptual diagram showing the effects of licorice on COVID-19 patients.

It is well established that, amongst other actions, SARS-CoV-2 infection becomes serious because of lung inflammation and multi-organ failure [196]. It is currently hypothesized that many lives could be saved if we could reduce the inflammation among seriously ill COVID-19 patients. The three key properties of licorice, its antiviral action, the autophagy enhancing mechanism, and its anti-inflammatory ability, might be able to improve the health status of COVID-19 patients in the following ways (Figure 5):

(i) If any system can block or inhibit the viral replication in the lung epithelial cells, it may be possible to prevent or reduce the body’s inflammatory response. Through several studies, it is found that the two triterpenes GL and GA, which are present in licorice, have significant antiviral characteristics against several viruses such as Hepatitis A, B, and C, HIV, Coxasackievirus, Influenza virus (H1N1 and H5N1), Duck Hepatitis virus, and SARS-CoV-1. In this respect, it is thus hypothesized that the GL and GA in licorice might prevent the spread of SARS-CoV-2, thereby significantly inhibiting any dangerous inflammatory response.
(ii) Chen and Du [197] conducted a molecular docking test recently and highlighted the potential binding of GL to the ACE-2 molecule. We already knew that SARS-CoV-2 enters the cell through ACE-2. Thus, blocking the ACE-2 by binding with GL can reduce the SARS-CoV-2 infectivity and the resulting COVID-19.
(iii) Autophagy is the suicidal mechanism that an infected cell adopts to protect adjacent cells from further infection., When SARS-CoV-2 infects a cell, the virus will forestall the autophagy mechanism by releasing SKP2 protein that can inhibit the cell’s Beclin-1 production. Whereas studies have shown that GL in licorice can increase the production of Beclin-1 by a factor of at least two times [107], which may help stimulate the autophagy mechanism and thus inhibit viral replication.
(iv) In the worst case, if the above two mechanisms cannot stop viral replication, there is the potentiality of inflammation in the body. In such a situation, adaptive immunity can be severely compromised, and this is where the phytochemicals in licorice, specifically GL and GA, can significantly reduce inflammation in the body by inhibiting the pro-inflammatory cytokines. In this way, licorice can possibly mitigate the severity of COVID-19 symptoms.

Licorice has a Federal Drug Administration (FDA) rating of ‘GRAS’ (Generally regarded as safe) [198]. Of interest is that during the first SARS-CoV epidemic in 2002–2003, an oral dose of 300 mg and an intravenous dose of 240 mg were recommended [199]. However, it was found that adverse reactions can result from overuse or overdose of licorice, including excessive Na ion levels and low K ions in the body. Such conditions lead to water retention and hypertension, respectively [127]. Heart disease related to excessive daily supplementation has also been reported [200]. It has been found that licorice supplementation can neutralize the intake of other medicines such as warfarin (Coumadin), a common anticoagulant agent [201,202]. In addition, it has been recommended that pregnant women limit their intake of licorice; as such, only a few conclusive studies have been conducted. Excessive long-term intake may (i) increase the levels of estrogen in the body and thus may lead to estrogen-mediated cancer and (ii) stimulate the anti-androgenic effect, which may lead to erectile dysfunction [141].

## 6. Conclusions

COVID-19 is spreading across the world with several thousand deaths every day and it is hitting many places as a secondary and tertiary waves. It is understood that if we can evade or alter ARDS, we can avoid the severity and mortality of most COVID-19 patients. A suitable antiviral and anti-inflammatory therapeutical intervention is required for this. Antiviral efficacy, anti-inflammatory property, and stimulation of the autophagy mechanism in cells are licorice properties which have a very high significance for COVID-19 patients. In addition, licorice-based phytochemicals have proven efficacy against a range of bacteria and fungi. Thus, it may be useful to avoid secondary bacterial infection in COVID-19 patients and treat other pathogenic diseases. An important corollary of this finding is that, because COVID-19 is of pandemic proportions, a plant-based medicine would be a boon for countries in the Afro-Asian-Pacific regions. Licorice is cheap and plentiful compared to modern allopathic medications, and it could thus make dramatic health improvements in the developing and underdeveloped world. Therefore, it is suggested that researchers should undertake in vitro and in vivo studies with GA and GL against SARS-CoV-2 and, based on the success, move forward with clinical trials, which may help mitigate COVID-19 severity.

## Figures and Tables

**Table 1:** A list of major bioactive compounds identified in licorice together with their therapeutical properties.

Bioactive Compounds	Properties	References
Glycerrhizin (GL)	Antimicrobial	[80,81,82]
18β-Glycerrhitinic acid	Antimicrobial, anti-inflammatory	
(GA)	Against *Helicobacter pylori*,	[83,84,85]
	MRSA,	
	Clarithromycin-resistant *H. pylori*	
18α-GC, 18β-GC	Anti-inflammatory	
Flavonoids (13 Ns below)	Anti-inflammatory	
Licochalcone A/B/C/D/E, isoliquiritigenin (ISL) echinatin (EC), glabridin (GLD), soangustone A (ISOA), licoricidin (LID), licorisoflavan A (LIA), dehydroglyasperin C (DGC), & dehydroglyasperin D (DGD) Glabridin	Antimicrobial, anti-inflammatory	[86]
	Antimicrobial	[87]
Aqueous extract	*B. Subtilis* and *E. Coli*	[88]
Methanol extract	Phytopathogenic fungi	[89]
Glycyrhetinic acid	MRSA	[84]
Licochalcone and Anti-fungal		[90]
Acetate root extract	*K. pneumonia* and *A. baylyi*	[91]
Glabron		
Licochalcone A/C/E	*Staphylococcus aureus*	[92]
Glycyrrhizin	*Helicobacter pylori*	[93]
18β-Glycerrhetinic acid	Clarithromycin-resistant *H. pylori*	[85]

GL is a glycoside formed as a mixture of Ca, Na, and K salts of glycyrrhizinic acid.

**Table 2:** A list of significant phytochemicals present in licorice and their antiviral efficacy.

Compounds in Licorice	Antiviral Property against	Reference
Glycyrrhizic acid	SARS-CoV	[111]
Glycyrrhizic acid derivatives	SARS-CoV	[110]
Glycyrrhizic acid	Hepatitis A (HAV)	[113]
Glycyrrhizic acid	Hepatitis B (HBV)	[81,114,115]
Glycyrrhizic acid	Hepatitic C virus	[101,102,116,117]
Glycyrrhizic acid	Human immune deficiency (HIV) Virus	[103,118,119,120]
Alkali root extract	HIV	[109]
Glycyrrhizic acid	Herpes viridae (varicella)	[121]
	Zoster virus (VZV)	[122]
	Epstien-Barr virus (EBV)	[122]
	Cytomegalovirus (CMV)	[123]
	Coxasackievirus B3 (CVB3)	[124]
	Coxasackievirus A16 (CVA16)	[57]
Glycyrrhizic acid	H5N1 influenza virus	[104,105,106]
	Duck Hepatitis virus	[108]
	Herpes simplex virus–1	[107]
		[125]
Water extract	HSV	[109]
18β-glycyrrhetinic acid	Rotavirus	[126]
